# One health in the Philippines: A review and situational analysis

**DOI:** 10.1016/j.onehlt.2024.100758

**Published:** 2024-05-22

**Authors:** Lystra Zyrill A. Dayapera, Jenica Clarisse Y. Sy, Sary Valenzuela, Samantha Julia L. Eala, Ciara Maria Ines P. Del Rosario, Karen Nicole C. Buensuceso, Adrian S. Dy, Danielle A. Morales, Anna Giselle Gibson, Geminn Louis C. Apostol

**Affiliations:** a SciLore LLC, Unit 10 Paz Madrigal Plaza, Madrigal Business Park, Ayala Alabang, Muntinlupa 1781, Metro Manila, Philippines; b Ateneo de Manila University School of Medicine and Public Health, Don Eugenio Lopez Sr. Medical Complex, Ortigas Ave, Pasig, Metro Manila 1604, Indonesia; c Department of Biology, School of Science and Engineering, Ateneo de Manila University, Katipunan Avenue, Loyola Heights, Metro Manila, Philippines; d Murdoch University, 90 South St, Murdoch, WA 6150, Australia

**Keywords:** One health, Philippines, One health coordination mechanism, Multisectoral coordination

## Abstract

The Philippines faces a complex and interconnected web of human, animal, and environmental health issues, including zoonotic and reverse zoonotic diseases, antimicrobial resistance, food insecurity and contamination, and threats from environmental degradation. This paper examines these issues, existing interventions, and their implementation challenges.

The overall framework used to analyze the level of operationalization of the One Health approach is the Multi-sectoral One Health Coordination Framework developed by the World Health Organization, Food and Agriculture Organization, and the World Organization for Animal Health. A two-step process was conducted: literature review, followed by consultations with government and non-government stakeholders across national, subnational, and local levels.

There has been significant progress in laying the foundation for collaboration between the human, animal, and environmental sectors. These are demonstrated by the presence of structures and systems, including inter-agency task forces, emergency response plans and mechanisms, and a network for health human resources. However, these are eclipsed by challenges, including the limited governance mechanisms within inter-agency committees, fragmented risk assessment and surveillance, untapped opportunities for joint investigation and response, insufficient resources for capacity-building, and absence of comprehensive risk communication and community engagement initiatives.

These challenges highlight the importance of promoting multi-sectoral governance and ensuring resource allocation and sharing. Joint activities across risk assessment, surveillance, investigation, and response are critical in ensuring a proactive and holistic approach to addressing threats. A well-capacitated interdisciplinary workforce, not only capable of managing these hazards but also empowering communities to protect themselves, is necessary in ensuring innovation and collaboration on health risks at the human-animal-environment interface.

In light of the multifaceted challenges faced by the Philippines, the One Health approach emerges as a vital strategy. By addressing governance issues, enhancing coordination, and bolstering resource allocation, the country can better protect the health and well-being of its people, animals, and ecosystems.

## Highlights

• The Philippines faces a complex and interconnected web of human, animal, and environmental health issues.
• There has been significant progress in operationalizing the One Health approach (i.e., governance mechanisms, response plans).
• Implementation challenges include fragmented risk assessment, surveillance, and insufficient capacity-building resources.
• There is a need to promote multi-sectoral governance, allocate resources, and implement joint activities across sectors.
• Strengthening the One Health approach in the Philippines is pivotal for better health outcomes and more effective responses to emerging threats.

## 1 Introduction

The Philippines is highly susceptible to disease outbreaks due to its geography, society, biodiversity, and culture. In 2019, it was categorized to be the most vulnerable to climate change impact in the world [1]. Factors such as population growth, migration, and limited access to clean resources contribute to the heightened spread of zoonotic diseases, vectors, and pathogens [2,3]. Population growth also drives encroachment, causing pollution, deforestation, habitat loss, and ecological disruptions that impact the welfare of both humans and animals [3]. Environmental degradation also affects the availability of ecosystem services, such as clean water and air, that support the wellbeing of humans and animals [4]. The COVID-19 pandemic has highlighted that current sectoral initiatives are inadequate to address these issues.

In the Philippines, there is an increase in efforts towards implementing a One Health approach to address issues that lie within the human-animal-environmental health interface. At the forefront of these initiatives is the establishment of Philippine Inter-Agency Committee on Zoonoses (PhilCZ), which is a One Health mechanism to strengthen the collaboration among the Department of Agriculture (DA), Department of Health (DOH), and Department of Environment and Natural Resources (DENR), to develop the national strategy on prevention, control, and elimination of zoonotic disease, and to ensure that the activities of the tripartite agencies are in line with the National Strategic Work Plan. While these departments endeavor to coordinate and collaborate on human-animal-environmental health issues, many issues in the systems contribute to the complexities and challenges of the One Health approach implementation in the Philippines, including a devolved health system. Agencies and departments often have their own agendas, plans, and budgets, which are reviewed and developed on different timelines. The implementation of these plans are also devolved to the regional and provincial local government units, who often have limited infrastructure, resources, and financial capacity, especially in the more rural regions [5].

Other interagency committees, such as the Inter-agency Committee on Antimicrobial Resistance (ICAMR) and Inter-agency Task Force on Zero Hunger have also been established, and national plans have also been developed to address different issues, such as AMR and biodiversity loss. The Joint External Evaluation (JEE) and State Party Self-Assessment Annual Report (SPAR) has been conducted to determine the current status of the country across different technical areas [6]. Most recently, the World Health Organization (WHO), World Organization for Animal Health (WOAH), and Food and Agriculture Organization (FAO) have supported collaborations across different sectors through the National Bridging Workshop (NBW). The workshop was used to identify gaps and areas for collaboration across the PhilCZ tripartite agencies to address problems concerning the animal, human, and environmental health sectors, respectively [7,8].

Despite this, there is a lack of comprehensive studies that systematically compiles and streamlines these efforts. Therefore, the significance of the proposed review lies in its role as a foundational step in setting a baseline and providing a holistic perspective of the One Health landscape in the Philippines. This review aims to summarize the current initiatives and the implementation challenges surrounding issues and programs in the Philippines that lie in the health, animal, and environmental nexus, from a One Health approach. Through a desk review and consultative process, this study aimed to 1) contextualize the nation for a better understanding of the conditions which may drive health risks, 2) outline initiatives that have been conducted in response to these emerging health threats, and 3) suggest goals for the nation to collaborate and accomplish in order to further the One Health agenda.

## 2 Methods

### 2.1 Phase 1: Desk review

A literature search and desk review of published (i.e. peer-reviewed journal articles) and grey literature (i.e. country assessments, policies, administrative orders, regulatory frameworks) were conducted in order to map out One Health issues in the Philippines from 2010 to 2023 according to the specific technical areas of the “Multisectoral Coordination Mechanisms Operational Tool - An operational tool of the Tripartite Zoonoses Guide”, the international guide for One Health planning and coordination by the published by WHO, FAO, WOAH [9]. This framework was used to assess the strengths, weaknesses, opportunities, threats, and succeeding recommendations of the One Health situation in the Philippines.

Electronic databases, specifically Google Scholar, PubMed, and Web of Science were used. Keywords used were: “One Health,” “zoonoses,” “antimicrobial resistance,” “disease surveillance,” biodiversity,” “vector borne diseases,” “food safety,” “environmental health,” “human health,” “animal health,” “agriculture,” “multisectoral,” “multisectoral approach”, “interdisciplinary collaboration.” Documents that did not contain specific provisions on One Health issues and those that have already been superseded by a national plan were excluded.

Current efforts from other governmental and non-governmental agencies were gathered, analyzed, and consolidated. National plans to address different issues, such as antimicrobial resistance (AMR) and biodiversity loss, were used as references. Information from the Philippines' JEE, SPAR, and NBW were used to determine the current status of the country across different technical areas.

These core documents were reviewed and summarized to identify the strengths, gaps, opportunities and threats for the operationalization of a One Health regional coordination mechanism. The results were validated through the consultative process. The final list included twenty one (21) reports, guidelines, and policy documents, summarized in Table 1.

**Table 1:** Summary of most-relevant initiatives and documents related to One Health in the Philippines.

Activity	Output	Year(s)	Reference
Finalization of the 2009 interim guidelines on leptospirosis	Philippine Clinical Practice Guidelines on the Diagnosis, Management and Prevention of Leptospirosis in Adults 2010	2010	(1)
The establishment of PhilCZ	Philippine Inter-Agency Committee on Zoonoses (PhilCZ)	2011	(2)
The implementation of RA 10536, “An Act Amending Republic Act No. 9296, otherwise known as ‘The Meat Inspection Code of the Philippines’”	Meat Inspection Code of 2013	2013	(3,4)
Food Safety Act of 2013	Republic Act (RA) 10,611	2013	(5)
The establishment of IATF-EID Development of EID Preparedness Manual for the prevention and control of EID	Inter-Agency Task Force for the Management of Emerging Infectious Diseases in the Philippines (IATF-EID)	2014	(6)
The establishment of the ICAMR	Inter-Agency Committee on Antimicrobial Resistance (ICAMR)	2014	(7)
Joint DA-DOH Administrative Order No. 2015–0007 implementing Rules and Regulations of the Food Safety Act of 2013.	Joint Administrative Order for the RA 10611	2015	(8)
Philippine Biodiversity Strategy and Action Plan	Philippine Biodiversity Strategy and Action Plan (PBSAP) 2015–2028	2016	(9)
The establishment of PhilRASAFF	Philippine Rapid Alert System for Food and Feed (PhilRASFF)	2017	(10)
Joint External Evaluation of IHR Capacities Republic of the Philippines Mission Report	PH Joint External Evaluation (JEE) Tool 2018	2018	(11)
Philippine Action Plan on Antimicrobial Resistance 2019–2023	Philippine National Action Plan on Antimicrobial Resistance 2019–2023	2018	(12)
The workshop developed a project proposal to operationalize OH approach in livestock systems	Workshop on Applications of OneHealth/EcoHealth Approach Towards Sustainable Livestock Production in Southeast Asia	2018	(13)
DOH Administrative Order No. 2018–0013 on the Revised Guidelines on the Management of Rabies Exposures	DOH AO No. 2018–0013	2018	(14)
The establishment of PhilOHUN	Philippine One Health University Network (PhilOHUN)	2019	(15)
Midterm review of the Malaria Strategic Plan 2017–2022	National Strategic Plan for the Control and Elimination of Malaria in the Philippines 2020–2022	2019	(16)
Meeting of the Inter-agency National ASF Task Force	DA Administrative Circular No. 12 s. Of 2019 on the National Zoning and Movement Plan for African Swine Fever	2019	(17)
Joint DOH-DA-DENR Administrative Order No. 2020–02	Joint DOH-DA-DENR Administrative Order No. 2020–02 on Guidelines to operationalize the Philippine Inter-agency Committee on Zoonoses (PhilCZ)	2020	(18)
National Rabies Prevention and Control Program Strategic Plan	National Rabies Prevention and Control Program 2020–2025	2020	(19)
National Food Safety Indicators workshop and concluding report	Development of National Food Safety Indicators with a One Health Approach	2020	(20)
The establishment of the Inter-Agency Task Force on Zero Hunger and The National Food Policy	Inter-Agency Task Force on Zero Hunger	2020	(21)
Executive Order No. 105 on the Creation of a National Task Force to prevent the entry of animal-borne diseases, contain and control the transmission thereof, and address issues relating thereto	E.O. No. 105 s. 2020	2020	(22)
National Strategic Plan for the Control and Elimination of Malaria in the Philippines 2020–2022	DOH Administrative Order 2021–0028 on the Implementing Guidelines on the Use of Online Malaria Information System (OLMIS)	2021	(23)
Introduction to One Health short course	Development of One Health Courses in the Philippines	2021	(24)
Philippines Disaster Management Reference Handbook	Philippines Disaster Management Reference Handbook	2021	(25)
Awareness campaign every year	Philippine Antimicrobial Awareness Week	2021	(26)
DOH Administrative Order No. 2021–0005	DOH Administrative Order No. 2021–0005 on the National Strategic Policy Framework for COVID-19 Vaccine Deployment and Immunization	2021	(27)
Philippine State Party Self-Assessment Annual Report	IHR State Party Self-Assessment Annual Report (SPAR) 2022	2022	(28)
National Bridging Workshop Roadmap and Report	National Bridging Workshop	2022	(29,30)
Regional short course	Introduction to One Health: Challenges and Opportunities to Achieve Better Public Health Outcomes	2022	(31)
Risk Communication Strategies Workshop and ASF Strategic Planning Meeting by the DA-BAI	The launch of the Bantay ASF sa Barangay (Babay) community-based approach.	2022	(32,33)
DA Memorandum Circular no. 49 series of 2023	DA Memorandum Circular No. 49 series of 2023 on the Guidelines on Targeted Vaccination As A Complementary Tool for the Control of Avian Influenza	2023	(34)
DOH Department Memorandum 2022–0526 on the Interim Guidelines on the Pilot-Implementation of the Integrated Sentinel Surveillance for SARS-CoV-2, SARI, and ILI	Establishment of a pan-respiratory surveillance for SARS-CoV-2, SARI, and ILI along with the conduct of the Training on Laboratory Procedures for Referral Laboratories by the Philippine National Influenza Center (PNIC) of the RITM	2023	(35,36)
1Health Project	National Inception Workshop for The One Human-Environment-Animal Linkage for Total Health (1HEALTH) Project on ASF, AI, and AMR Surveillance	2023	(37)
Interim guidelines for collection, handling, processing, packaging and transport of specimens from Nipah Suspected Cases	Interim guidelines for collection, handling, processing, packaging and transport of specimens from Nipah Suspected Cases	2023	(38)
Overall assessment of environmental sector in preparation for One Health Joint Plan of Action 2022–2026	Environmental Health Needs Assessment and Validation Workshop	2023	(39)
2023–2028 National Strategic Plan on Zoonoses	Planning and Review Workshop on the 2023–2028 National Strategic Plan on Zoonoses	2023	(40)
National Action Plan for Health Security (NAPHS)	PhilCZ Workshop for NAPHS	2023	(41)
FDA establishes Task Force to focus on the evaluation of HPAI vaccines	Establishment of Task Force Alectryon	2023	(42)

### 2.2 Phase 2: Consultation phase

#### 2.2.1 Key-informant interviews

Relevant national, subnational, and local stakeholders, who were involved or affected by the projects listed in Table 1, were identified and mapped through consultative, in-depth key-informant interviews. Key personnel from the lead member agencies of the PhilCZ were consulted. The key informants selected were from government agencies such as the DOH Epidemiology Bureau (EB), DOH Pharmaceutical Division (PD), DENR Biodiversity Management Bureau (BMB), DA Bureau of Animal Industry (BAI), and DOH Office of Health Laboratories (OHL), as well as consultants from United States Agency for International Development International Health Regulations (USAID IHR). A total of seven key-informant interviews were conducted. These interviews were recorded and transcribed, then underwent deductive thematic manual analysis.

#### 2.2.2 Workshop with key government and non-government stakeholders

Two technical vetting workshops with key stakeholders to discuss the status and challenges of the implementation of the One Health approach. Internal focal persons and resources were identified at the national and subnational level, including the academe, civil society, and private sector.

The first workshop was conducted at the Ateneo School of Medicine and Public Health (ASMPH), Pasig City, on 27 July 2023, which had a total of thirteen (13) participants from seven (7) bureaus and organizations. The second grand stakeholder workshop was conducted at the Luxent Hotel, Quezon City on 3 August 2023 with 53 attendees from 29 national bureaus and organizations.

A participatory vetting workshop design among the attendees was conducted. Participants were split into groups per sector to vet the issues, strategies, and future directions drafted by the team. Each thematic point was raised by a moderator, so each group reported their shared insights to all attendees for further discussion. From all participants, consents were obtained and confidentiality and privacy were maintained.

## 3 Results

The findings of the level of institutionalization and operationalization of one health at the national levels were summarized using the framework provided by the “Multisectoral Coordination Mechanisms Operational Tool - An operational tool of the Tripartite Zoonoses Guide” published by WHO, FAO, and WOAH in 2022. The framework outlining the application of Multisectoral One Health coordination in the following technical activities, as defined and summarized in Table 2.

a. Governance, Policy, and Financing

**Table 2:** Definition and Components of the Multisectoral One Health coordination framework.

Technical Activity	Definition	Key achievements/Progress
1. Governance, Policy and Financing	Initiatives towards the integration of one health principles in developing and reinforcing legislation and policies; and to optimize resource allocation for One Health through innovative institutional mechanisms.	• Establishment of the Philippine Inter-Agency Committee on Zoonoses (PhilCZ) • Establishment of the Inter-Agency Task Force for the Management of Emerging Infectious Diseases in the Philippines (IATF-EID) • Establishment of the Inter-Agency Committee on Antimicrobial Resistance (ICAMR) • Establishment of the Inter-agency Committee on Environmental Health (IACEH) • Establishment of the Inter-Agency Task Force on Zero Hunger • Allocation of Php 2 million annually for the operations of the PhilCZ • Program managers monitor, review, and assess policies through program implementation reviews, leading to policy revisions based on the findings from these reviews and other pertinent research. • Allocation of funds from various sources, including the national budget specified in the General Appropriations Act at both national and sub-national levels, contributions from National and Local Disaster Risk Reduction and Management Councils, Local Government Units dedicating 5% of their internal revenue allotment (70% for disaster-related purposes and 30% for Quick Response Funds), and assistance from the Philippine Health Insurance Corporation (PhilHealth) through benefit packages for infectious diseases and standard healthcare services, supports the implementation of IHR (2005).
2. Joint Risk Assessment, Surveillance and, Information Sharing	Initiatives that aim to identify potential health threats among different agencies by assessing and analyzing risks across different sectors and developing and implementing preparedness plans and system for rapid response	• Establishment of the Philippine Integrated Disease Surveillance and Response (PIDSR) as an indicator-based surveillance system for notifiable human diseases • Establishment of the Philippine Rapid Alert System for Food and Feed (PhilRASFF) • Development of the National Disaster Risk Reduction and Management Plan (NDRRMP), National Framework Strategy on Climate Change 2010–2022, and Disaster Management Reference Handbook • Development of Event-Based Surveillance and Response System (ESR) • Notifiable zoonotic disease testing is directed to Regional Animal Disease Diagnostic Laboratories (RADDL) • Monitoring of meat samples by NMIS • Establishment of passive surveillance through the Wildlife Disease Surveillance Conceptual Framework, National Focal Point for Animal Disease Notification, and DOH when necessary
3. Investigation and Response	Initiatives involving the development and implementation of response plans, mobilizing resources, and coordinating multi-sectoral efforts to investigate and respond to outbreaks and threats.	• Inter-agency collaboration through the ICAMR, PhilCZ, NDRRMC, Food Safety Regulation Coordinating Board (FSRCB), University of the Philippines Los Banos (UPLB) Program for Zoonotic Diseases, and Inter-Agency Task Force for the Management of Emerging Infectious Diseases in the Philippines (IATF-EID) • EB was designated as the National IHR Focal Point (NFP) notifying the WHO of relevant health events through a weekly International Health Event report • Development of National Food Safety Indicators with a One Health Approach • Development of the Philippine National Action Plan on Antimicrobial Resistance 2019–2023 • National Rabies Prevention and Control Program Strategic Plan 2020 • Establishment of a National Technical Working Group, and mandatory IPC licensing for hospitals • Zoonotic diseases are addressed through the activities of PhilCZ and the Emerging and *Re*-emerging Infectious Disease (EREID) program. • Simulation exercises and the active participation of the DOH were instrumental in preventing human cases during the 2017 Highly Pathogenic Avian Influenza (HPAI) outbreak in poultry.
4. Planning and Preparedness	Initiatives to develop and implement preparedness plans for one health; including simulations and training exercises, building capacity for outbreak response, and establishing mechanisms for rapid response.	• Assessment of the Philippines using the Joint External Evaluation (JEE) Tool in 2018 and preparation for a re-assessment scheduled for 2024 • Establishment of the National Disaster Preparedness Plan 2015–2028, multi-hazard response plans, the Manual of Operations and SOPs for Health Emergency and Disaster Response Management, as well as the Philippine Avian Influenza Protection Program. • Research programming on field and on-farm disease investigation, surveillance, and control management has been done through The 1Health Project (“1Health”) • Launching of the first Disaster Risk Reduction Management in Health (DRRM-H) Center in 2022 through UPM
5. Workforce Development	Initiatives to build the capacity of health workers and other stakeholders to prevent, detect, and respond to One Health threats; including the development of training programs and promoting collaboration among different sectors.	• Development of the Philippine Field Epidemiology Training Program (PFETP) 1987 • Establishment of the Human Resources for Health (HRH) Network of the Philippines 2006 • Veterinary services are supported by training from the BAI, Agricultural Training Institute (ATI) and LGUs 2017? • Establishment of the Philippine One Health University Network (PhilOHUN) 2019 • Implementation of the Human Resource for Health Deployment Program to redistribute the healthcare personnel in the country 2019 • Development of the Learning and Development Plan and National Human Health Resources for Health Master Plan 2020–2040 2020 • Development of One Health Courses in the Philippines 2021 • Creation of competency development programs such as Environment and Natural Resources Academy (ENRA) courses for DENR employees. 2022 • Establishment of One Health Fellowships, such as the Next Generation One Health Fellowship (est. 2022 by Saint Luke's Medical Center College of Medicine - local), the ASEAN-Australia One Health Fellowship (est. 2023 by Murdoch University - regional), and Southeast Asian One Health University Network (SEAOHUN) One Health Fellowship. • Established biosafety and biosecurity training modules at institutional levels, including RITM and the University of the Philippines, while various private organizations have conducted training in biosafety and biosecurity. • National laboratory referral systems provide training and SOPs for packaging and transporting laboratory specimens.
6. Risk Communication	Initiatives towards implementing transparent risk communication strategies to inform and engage the public and other stakeholders about one health risks and impacts	• Establishment of the Health Promotion Bureau (HPB), which aims to promote health education, and the Communication (COM) Office, which takes charge in the risk communication plans of the DOH during times of crisis. • A risk communication plan is developed per program for the DA & DENR • Inter-departmental collaboration for risk communication on African Swine Fever (ASF)

The results of the study have been summarized in the table below according to strengths, weaknesses, opportunities, and threats (SWOT) analysis in Table 3.

**Table 3:** SWOT analysis outcome on Multisectoral One Health coordination framework.

Technical Activity	Strengths	Weaknesses	Opportunities	Threats
1. Governance, Policy and Financing	Established inter-agency task forces (i.e. PhilCZ, IACEH). Mandated Php 2 million annually for the operations of the PhilCZ and Php 1 million for emergency funds from the Office of the President.	Existing governance mechanisms rely on the capacities of personnel drawn from various departments, resulting in excessive workload and disjointed approach in the conduction and implementation of PhilCZ activities. Inadequate funding for capacity-building activities, such as trainings, resources, and workforce development.	Formal roles have still remained undefined, especially in the sub-national level. No formalized multi-sectoral policy instruments for the joint implementation of some disease programs such as the Philippine Animal Disease Profiling Program, the continuous monitoring of zoonotic diseases at points of entry, and biosafety and biosecurity protocols. Creation of new legislation, such as the “One Health Act.” Organize exercises and after-action reviews to ensure lessons learned are used to update relevant procedures and plans. Inclusion of memberships of the non-tripartite government agencies that have relevant roles in One Health (i.e. Department of the Interior and Local Government (DILG), Department of Public Works and Highways (DPWH), Department of Finance (DOF), National Economic and Development Authority (NEDA), Department of Science and Technology (DOST)). Institutionalize a list of priority diseases under the purview of the PhilCZ through a Joint Administrative Order. Create One Health Response Teams at the national, regional and local levels and Regional Committees on Zoonosis (RCZs) with Technical Working Groups (TWGs). Establish more sustainable financial mechanism for One Health activities and emergency contingency funding to procure and maintain national stockpiles such as stockpiles for personal protective equipment (PPE), antivirals, vaccines, and other emergency supplies and equipment. Creation of a budget tracking and monitoring framework for regular sources of funding.	Funding of activities is subject to budgetary appropriations of each department through the annual budget proposals. National departments have separate agendas and action plans. AMR is currently not included in the DENR priority programs, therefore creating difficulties in securing funding and resources for related initiatives.
2. Joint Risk Assessment, Surveillance and, Information Sharing	Well-developed disaster response plans and mechanisms (i.e. National Disaster Risk Reduction and Management Plan (NDRRMP), National Framework Strategy on Climate Change 2010–2022, and Disaster Management Reference Handbook). Establishment of the PIDSR, which serves as an indicator-based surveillance system in the DOH. Event-Based Surveillance and Response System (ESR), spearheaded by the Epidemiology Bureau, utilizes online electronic reporting and real-time reporting within the regional health divisions.	Minimal joint risk assessments conducted involving a One Health approach. Surveillance of animal, health, and environmental diseases are handled by separate departments. Reporting delays between local and national levels, and information feedback delays from the national level to regional and city/provincial levels.	Different departments are now currently under preparations for the development of the National Action Plan on Health Security and the 2024 JEE. Perform collaborative multi-sectoral risk assessments at all levels. Enhance collaboration, strengthen networks, and promote coordination among relevant stakeholders, including government agencies, healthcare institutions, research organizations, and other community representatives. Establish a regulatory system inclusive of the private and government laboratories handling high-consequence biological agents. Increase sampling coverage, optimizing systems, guidelines, and methodologies for health emergency assessments. Develop and maintain a national operational response plan for public health emergencies with adequate workforce development. Periodic review and evaluation of new technologies should be conducted to evaluate if these can be integrated or adopted to improve existing systems. Establish a A Joint One Health National Disease Surveillance, Prevention, and Control System. Strengthen data management through the introduction of a unique case identification system and enhance the coordination and information sharing between human and animal/wildlife surveillance. Strengthen the timeliness and completeness of both indicator- and event-based surveillance systems. Improve data protection and sharing by implementing a secure electronic tool for laboratory data transfer, reconciling data from multiple sources, and centralizing notifiable diseases in a single electronic platform. Enhance animal/wildlife health surveillance by improving reporting and information sharing with human health surveillance at all levels.	Most surveillance systems for zoonotic diseases operate on an event-based model, with limited active monitoring of antimicrobial resistance (AMR) within the animal sector. This approach may overlook instances of AMR occurrence.
3. Investigation and Response	The DOH EB, as the designated National IHR Focal Point (NFP) of the Philippines, demonstrates constant communication with the WHO and DA-BAI, notifying the organization of relevant health events. Formulation of interagency collaborations and task forces. Establishment of national plans to investigate One Health issues.	Outside the DOH and apart from BAI, there is a lack of coordination and communication between the NFP and other agencies. Limited scope and coverage in investigation and response activities.	Opportunity to develop a national Standard Operating Procedure (SOP) discussing information sharing, field investigation, response, sharing of logistics/resources for food safety incidents, outbreaks, and emergencies. Strengthen the referral system for laboratories.	Limited resources, capacity, and infrastructure for prevention, detection and management of zoonotic diseases.
4. Planning and Preparedness	Well-developed plans for certain One Health issues (i.e., National Disaster Preparedness Plan 2015–2028, multi-hazard response plans, the Manual of Operations and SOPs for Health Emergency and Disaster Response Management, Philippine Avian Influenza Protection Program). Planning and action research programming on field and on-farm disease investigation, surveillance, and control management has also been done through The 1Health Project. The First Disaster Risk Reduction Management in Health (DRRM-H) Center seeks to equip emergency responders and local government units with the appropriate skills and knowledge through simulation, as well as by providing tools.	Capacity assessment and resource mapping are mainly conducted at the national and regional levels.	Highlight inter-agency coordination and legislation at all levels during development of national plans based on the current strategic risk assessment findings Build capacity for outbreak response. Strengthen systems, services, and monitoring capabilities to detect, respond to, and mitigate health threats effectively. Enhance health emergency risk assessments with a focus on health risks, impacts, and system vulnerabilities. Allocate resources for public health emergency preparedness based on local needs and risk assessment. Utilize post-action reviews of health emergency responses to update emergency preparedness and response legislation, procedures, and plans. Apply lessons from disaster health responses to develop international reference guidelines for enhancing domestic medical countermeasures.	Limited laboratory capacity. Lack of exercises including medical countermeasures for emergency events, especially at subnational levels. Insufficient documentation and post-event reviews for disease outbreaks.
5. Workforce Development	Establishment of the Human Resources for Health (HRH) Network of the Philippines. Establishment of the Human Resource for Health Deployment Program to redistribute the healthcare personnel in the country. Creation of One Health courses and further education/training programs.	Insufficient manpower to conduct all workforce development initiatives.	Capacitate data analysis and quality management skills across all sectors. Conduct One Health training programs towards detecting, reporting, and assessing diseases including joint trainings on risk communication, deploying Management Teams, and managing logistics at all levels. Establish a more robust governance mechanism for the Human Resource for Health Network to oversee the inclusion, monitoring, and tracking of the multi-sectoral public health workforce.	Need to build technical skills of personnel. Lack of awareness and understanding for “One Health.” Lack of technical capacity to mount joint efforts across agencies (ie. agencies do not know how to do joint risk assessment or joint responses).
6. Risk Communication	Establishment of Communication (COM) Officeto handle the risk communication plans of the DOH. Inter-departmental collaboration for risk communication on certain diseases (i.e. African Swine Fever).	Lack of coordination mechanism and established formalized commitment of partner sectors. Lack of robust risk communication plan/MOU/guidance for community engagement across all agencies and partners.	Need for a more robust risk communication training and plan for community engagement across the different sectors. Develop a framework for routine evaluation of planned risk communication interventions and testing of operational mechanisms.	Lack of sufficient funding and personnel for a risk communication plan at all levels.

1. Philippine Society for Microbiology and Infectious Diseases. Clinical Practice Guidelines for Leptospirosis 2010 [Internet]. 2010. Available from: https://www.psmid.org/cpg-leptospirosis-2010/

2. President of the Philippines. CREATING THE PHILIPPINE INTER-AGENCY COMMITlEE ON ZOONOSES, DEFINING ITS POWERS, FUNCTIONS, RESPONSIBILITIES, OTHER RELATED MATTERS AND PROVIDING FUNDS THEREOF [Internet]. Administrative Order No. 10 Apr 11, 2011. Available from: https://www.officialgazette.gov.ph/downloads/2011/04apr/20110411-AO-0010-BSA.pdf

3. Republic Act 10536 An Act Amending Republic Act No. 9296, Otherwise Known as “The Meat Inspection Code of the Philippines” [Internet]. Jul 23, 2012. Available from: https://www.officialgazette.gov.ph/downloads/2013/05may/20130515-RA-10536-BSA.pdf

4. Department of Agriculture. Administrative Order No. 09 series of 2013 on the Supplemental Guideline to DA A.O. No. 26 series of 2005, Section III: Accreditation Procedure for Meat Importers [Internet]. 2013. Available from: https://nmis.gov.ph/images/pdf/ao-09-2013.pdf

5. Republic of the Philippines. Republic Act No. 10611 Food Safety Act of 2013 [Internet]. Jul 23, 2012. Available from: https://icrs.gcg.gov.ph/files/ZwEcCjCRzf50gA3bTJnn.pdf

6. President of the Philippines. Executive Order No. 168 Creating the Inter-agency Task Force for the Management of Emerging Infectious Diseases in the Philippines [Internet]. May 26, 2014. Available from: https://www.officialgazette.gov.ph/downloads/2014/05may/20140526-EO-0168-BSA.pdf

7. President of the Philippines. Administrative Order No. 42 Creating an Inter-agency Committee for the Formulation and Implementation of a National Plan to Combat Antimicrobial Resistance in the Philippines [Internet]. Apr 10, 2014. Available from: https://www.officialgazette.gov.ph/downloads/2014/04apr/20140410-AO-0042-BSA.pdf

8. Department of Agriculture, Department of Health. Joint DA-DOH Administrative Order No. 2015-2007 THE IMPLEMENTING RULES AND REGULATIONS OF REPUBLIC ACT NO. 10611, “AN ACT TO STRENGTHEN THE FOOD SAFETY REGULATORY SYSTEM IN THE COUNTRY TO PROTECT CONSUMER HEALTH AND FACILITATE MARKET ACCESS OF LOCAL FOODS AND FOOD PRODUCTS, AND FOR OTHER PURPOSES” OTHERWISE KNOWN AS THE “FOOD SAFETY ACT OF 2013.” [Internet]. Feb 20, 2015. Available from: https://faolex.fao.org/docs/pdf/phi154130.pdf

9. Philippines, editor. Philippine biodiversity strategy and action plan, 2015-2028: bringing resilience to Filipino communities. Diliman, Quezon City: Department of Environment and Natural Resources, Biodiversity Management Bureau; 2016. 248 p.

10. PhilRASFF. What is the Philippine Rapid Alert System for Food and Feed (PhilRASFF)? [Internet]. Available from: https://philrasff.fda.gov.ph/

11. World Health Organization. JOINT EXTERNAL EVALUATION OF IHR CORE CAPACITIES of the REPUBLIC OF THE PHILIPPINES [Internet]. 2018 Sep. Available from: https://extranet.who.int/sph/sites/default/files/document-library/document/JEE%20Report%20Republic%20of%20the%20Philippines%202018.pdf

12. Inter-Agency Committe on Antimicrobial Resistance. The Philippine Action Plan to Combat Antimicrobial Resistance 2019-2023 [Internet]. Available from: https://cdn.who.int/media/docs/default-source/antimicrobial-resistance/amr-spc-npm/nap-library/philippine-national-action-plan-on-amr-2019-2023-final.pdf?sfvrsn=8bbe1fdb_1&download=true

13. School of Environmental Science and Management. University of the Philippines Los Banos. 2018. SESAM Co-organizes Workshop on Onehealth/Ecohealth Towards Sustainable Livestock Production in the Philippines and Southeast Asia. Available from: https://sesam.uplb.edu.ph/news/sesam-co-organizes-workshop-on-onehealth-ecohealth-towards-sustainable-livestock-production-in-philippines-and-southeast-asia/

14. Department of Health Office of the Secretary. Administrative Order No. 2018-0013 Revised Guidelines on the Managment of Rabies Exposures [Internet]. Apr 16, 2018. Available from: https://www.psmid.org/revised-guidelines-on-the-management-of-rabies-exposures-ao-2018-0013/

15. PHILOHUN. Philippine One Health University Network [Internet]. Available from: https://www.philohun.org/

16. Department of Health. National Strategic Plan for the Control and Elimination of Malaria in the Philippines 2020-2022 [Internet]. 2020. Available from: https://www.apmen.org/sites/default/files/all_resources/Philippines_National%20Strategic%20Plan_2020-2022.pdf

17. Department of Agriculture. Administrative Circular No. 12 Series of 2019 National Zoning and Movement Plan for the Prevention and Control of African Swine Fever [Internet]. Dec 10, 2019. Available from: https://livestock.da.gov.ph/wp-content/uploads/2021/11/ac12_s2019.pdf

18. Department of Health, Department of Agriculture, Department of Environment and Natural Resources. Joint DOH-DA-DENR Administrative Order No. 2020-02 Guidelines to operationalize the Philippine Inter-agency Committee on Zoonoses (PhilCZ) [Internet]. 2020. Available from: https://www.da.gov.ph/wp-content/uploads/2020/11/doh_da_denr_s2020.pdf

19. Department of Health, Department of Agriculture. National Rabies Prevention and Control Program 2020-2025 [Internet]. 2020. Available from: https://rr-asia.woah.org/wp-content/uploads/2020/03/final-mtp-rabies_philippines.pdf

20. Food and Agriculture Organization. Concluding workshop of the Pilot Project on National Food Safety Indicators in the Philippines Workshop report [Internet]. 2020. Available from: https://www.fao.org/3/ca9713en/ca9713en.pdf

21. President of the Philippines. Executive Order No. 101 Creating an Inter-agency Task Force on Zero Hunger [Internet]. Jan 10, 2020. Available from: https://www.officialgazette.gov.ph/downloads/2020/01jan/20200110-EO-101-RRD.pdf

22. PRESIDENT OF THE PHILIPPINES. Executive Order No. 105, s. 2020 CREATING A NATIONAL TASK FORCE TO PREVENT THE ENTRY OF ANIMAL-BORNE DISEASES, CONTAIN AND CONTROL THE TRANSMISSION THEREOF, AND ADDRESS ISSUES RELATING THERETO [Internet]. Feb 21, 2020. Available from: https://www.officialgazette.gov.ph/2020/02/21/executive-order-no-105-s-2020/

23. Department of Health Office of the Secretary. Administrative Order No. 2021-0028 Implementing Guidelines on the Use of Online Malaria Information System (OLMIS) [Internet]. 2021. Available from: https://law.upd.edu.ph/wp-content/uploads/2021/06/DOH-Administrative-Order-No-2021-0028.pdf

24. Angeles K, Boc GG, Co S, Fowler KC, Hidalgo BY, Mendoza JPD, et al. Bridging the Knowledge Gap: A Biosecurity Research Agenda for the Philippines. 2023 [cited 2024 Apr 15]; Available from: https://www.ssrn.com/abstract=4656278

25. 2021 Philippines Disaster Management Reference Handbook. Relief Web [Internet]. 2021 Nov 22; Available from: https://reliefweb.int/report/philippines/2021-philippines-disaster-management-reference-handbook

26. Pharmaceutical Division. GovPH. 2021. PHILIPPINE ANTIMICROBIAL AWARENESS WEEK 2021 – PRESS RELEASE. Available from: https://pharma.doh.gov.ph/2021/11/18/philippine-antimicrobial-awareness-week-2021-press-release/

27. Department of Health Office of the Secretary. Administrative Order No. 2021-0005 National Strategic Policy Framework for COVID-19 Vaccine Deployment and Immunization [Internet]. 2021. Available from: https://law.upd.edu.ph/wp-content/uploads/2021/05/DOH-Administrative-Order-No-2021-0005.pdf

28. e-SPAR [Internet]. Electronic IHR States Parties Self-Assessment Annual Reporting Tool. Available from: https://extranet.who.int/e-spar/

29. NATIONAL BRIDGING WORKSHOP ROADMAP (AUGUST 2022) FOR THE PHILIPPINES [Internet]. 2022. Available from: https://extranet.who.int/sph/sites/default/files/document-library/document/NBW%20Philippines%20-%20Final%20Roadmap.pdf

30. Department of Health, Department of Agriculture, Department of Environment and Natural Resources, World Health Organization, World Organisation for Animal Health, Food and Agriculture Organization. National Bridging Workshop on the International Health Regulations (IHR) and the Performance of Veterinary Services (PVS) Pathway [Internet]. 2022. Available from: https://extranet.who.int/sph/sites/default/files/document-library/document/NBW%20Philippines%20-%20Final%20Report.pdf

31. Universirt of the Philippines Alumni [Internet]. Introduction to One Health: Challenges and Opportunities to Achieve Better Public Health Outcomes. Available from: https://alum.up.edu.ph/event/introduction-to-one-health-challenges-and-opportunities-to-achieve-better-public-health-outcomes/

32. Masilang JP. Department of Agriculture - Agricultural Training Institute. 2022. ASF Prevention Campaign Players Convene to Boost Readiness, Efforts. Available from: https://ati2.da.gov.ph/ati-main/content/article/jayvee-p-masilang/asf-prevention-campaign-players-convene-boost-readiness-efforts

33. Llanes J. DA-BAI conducts strategic planning to improve ASF control measures. Herald Express [Internet]. 2022 Dec 14; Available from: https://baguioheraldexpressonline.com/da-bai-conducts-strategic-planning-to-improve-asf-control-measures/

34. Department of Agriculture. DA Memorandum Circular No. 49 series of 2023 on the Guidelines on Targeted Vaccination As A Complementary Tool for the Control of Avian Influenza [Internet]. 2023. Available from: https://apps.fas.usda.gov/newgainapi/api/Report/DownloadReportByFileName?fileName=The%20Philippines%20Issues%20Guidelines%20for%20Avian%20Influenza%20Vaccination_Manila_Philippines_RP2023-0068.pdfhttps://apps.fas.usda.gov/newgainapi/api/Report/DownloadReportByFileName?fileName=The%20Philippines%20Issues%20Guidelines%20for%20Avian%20Influenza%20Vaccination_Manila_Philippines_RP2023-0068.pdf

35. Dimaano AA. Research Institute for Tropical Medicine. 2023. DOH integrates ILI, SARI, SARS-CoV-2 surveillance operations. Available from: https://ritm.gov.ph/pnicstakeholdersmeeting/

36. World Health Organization. Philippines- Coronavirus Disease 2019 (COVID-19) Situation Report #138 [Internet]. 2019. Available from: https://cdn.who.int/media/docs/default-source/wpro---documents/countries/philippines/emergencies/covid-19/who-phl-covid-19-situation-report-138.pdf?sfvrsn=bb7d4d0_1

37. School of Environmental Science and Management. University of the Philippines Los Banos. 2023. UPLB-SESAM holds the National Inception Workshop for The 1HEALTH Project on African Swine Fever, Avian Influenza, and Antimicrobial Resistance Surveillance. Available from: https://sesam.uplb.edu.ph/news/uplb-sesam-holds-the-national-inception-workshop-for-the-1health-project-on-african-swine-fever-avian-influenza-and-antimicrobial-resistance-surveillance/

38. Research Institute for Tropical Medicine. INTERIM GUIDELINES FOR COLLECTION, HANDLING, PROCESSING, PACKAGING AND TRANSPORT OF SPECIMENS FROM NIPAH SUSPECTED CASES [Internet]. 2023. Available from: https://ritm.gov.ph/interim-guidelines-for-collection-handling-processing-packaging-and-transport-of-specimens-from-nipah-suspected-cases/

39. World Health Organization, Food and Agriculture Organization of the United Nations, World Organisation for Animal Health, United Nations Environment Programme. World Health Organization. 2022. One health joint plan of action (2022–2026): working together for the health of humans, animals, plants and the environment. Available from: https://www.who.int/publications/i/item/9789240059139

40. Program for Zoonotic Diseases. University of the Philippines Los Banos. UPLB PZD FACILITATES PHLCZ PLANNING AND REVIEW WORKSHOP ON THE 2023-2028 NATIONAL STRATEGIC PLAN ON ZOONOSES. Available from: https://zoonoses.uplb.edu.ph/news/uplb-pzd-facilitates-phlcz-planning-and-review-workshop-on-the-2023-2028-national-strategic-plan-on-zoonoses/

41. World Health Organization. National Action Plan for Health Security (NAPHS) and Costing [Internet]. 2024. Available from: https://extranet.who.int/sph/naphs

42. Espina ZE. FDA launches task force to help combat avian flu threat. 2023 Dec 28; Available from: https://mb.com.ph/2023/12/28/fda-launches-task-force-to-help-combat-avian-flu-threat

Due to the necessity for a multisectoral approach, the government has established different groups, such as the PhilCZ, ICAMR, Inter-Agency Task Force for the Management of Emerging Infectious Diseases (IATF-EID), and Inter-agency Committee on Environmental Health (IACEH). The PhilCZ, composed of agencies under the DOH, DA, and DENR, is an organization that came into existence by Administrative Order No. 10, signed in 2011. It is a One Health mechanism to entail strengthened collaboration between animal-human health and environment sectors for the prevention and control of zoonoses at the local and national levels [10]. According to the Administrative Order, each department is mandated to allocate Php 2 million annually for the operations of the PhilCZ. The ICAMR was formed as a government response for the need to regulate AMR through the formulation, adoption, and implementation of a comprehensive national plan [11]. The members of the ICAMR spearhead the surveillance of AMR and promote the rational use of antimicrobials in the human and animal health sides. While the DENR is included in AMR discussions, they have yet to be formally included as a PhilCZ member. Funding of activities by the committee is subject to the budgetary appropriations of each department, and through the annual budget proposals through the General Appropriations Act (GAA). The IACEH aims to protect the environment by formulating policies and developing programs for environmental health protection [12]. There is Php 1 million allocated from the emergency funds of the Office of the President to carry out activities of the IACEH. Subsequent budget is incorporated in the GAA under the DOH. The IATF-EID was created through Executive Order No. 168 in 2014 to assess, monitor, control, and prevent emerging infectious diseases that could progress into a pandemic [13]. In 2020, they spearheaded the creation of the National Action Plan against COVID-19 [14]. Currently, the DENR is also not a member of this multi-sectoral task force.

b. Joint Risk Assessment, Surveillance, and Information Sharing

Risk assessment plays a vital role in safeguarding both human and animal health. Different agencies in the Philippines have designed various initiatives which aim to identify and mitigate various different health threats across the country. Some efforts on risk assessment include the National Disaster Risk Reduction and Management Plan (NDRRMP) [15], National Framework Strategy on Climate Change 2010–2022 [16], and Disaster Management Reference Handbook [17], among others. However, at present, there has only been minimal joint risk assessments conducted involving a One Health approach, such as the conduct of the fifth stakeholders' forum on the control and elimination of neglected tropical diseases (NTDs) [18], and the 2018 JEE. Although, the different departments are now currently under preparations for the development of the National Action Plan on Health Security and the 2024 JEE.

The Philippine Integrated Disease Surveillance and Response (PIDSR) serves as an indicator-based surveillance system in the DOH [19]. Syndromic surveillance systems for acute bloody diarrhea, acute hemorrhagic fever, acute meningoencephalitis syndrome, severe acute respiratory infection, and influenza-like illness are also in place. Government health facilities are assigned to collate all of the data gathered. The Province/City Epidemiology and Surveillance Unit refines the data gathered and sends it to the Regional Epidemiology and Surveillance Units. This institute compiles the data and sends it to the DOH-EB. Furthermore, the Research Institute for Tropical Medicine (RITM), which spearheads a network of 26 laboratories/sentinel sites, contributes laboratory-based data, whether these be general or case-to-case. They also collate data from their network to support the Antimicrobial Resistance Surveillance Program of the country. In the case of event-based surveillance, the Event-Based Surveillance and Response System (ESR) supervised by the EB authorizes every administrative level and relevant offices to record public health events. The ESR utilizes online electronic reporting and real-time reporting within the health division [19].

The DA handles surveillance with regards to reports on domestic animals and meat establishments. Field reports of notifiable zoonotic diseases in domestic animals are directed to municipal or provincial veterinary offices and undergo testing through the Regional Animal Disease Diagnostic Laboratories (RADDL). On the other hand, the National Meat Inspection Service (NMIS) handles the monitoring of meat samples. The BAI is notified of the results of both types of investigation. As previously mentioned, the Philippine Animal Health Information System (Phil-AHIS) also serves as a surveillance and information sharing system within the DA. It gathers all essential information regarding disease surveillance, immunization, and the relocation of animals in the event of an outbreak of any related illness within the nation [20,21].

Surveillance in the DENR has been established through the Wildlife Disease Surveillance Conceptual Framework, which was created by the BMB in 2018 [22]. The framework serves as the DENR's approach to monitor diseases in the context of One Health. Based on recent reports, mostly passive surveillance activities have been conducted. This surveillance system is supported by the 10.13039/501100003582BAI, and the BAI Animal Disease Diagnostic and Reference Laboratory (ADDRL). The BAI and ADDRL serve as testing sites that examine samples collected by the BMB from wildlife through the catch and release program. Laboratory results are reported to the BAI, the National Focal Point for Animal Disease Notification, and DOH when deemed necessary. The Joint Administrative Order 2020–02 presents a communication and collaboration mechanism of the PhilCZ with regards to surveillance and information sharing, but is limited to zoonotic diseases [23].

c. Investigation and Response

Various policies, initiatives, and programs have been established to facilitate a multi-sectoral approach to investigating and responding to outbreaks and threats in the country. The EB, designated as the designated National IHR Focal Point (NFP) of the Philippines, demonstrates constant communication with the WHO, notifying the organization of relevant health events. The NFP distributes a weekly International Health Event report based on the Event Information Site (EIS) to specific bureaus within the DOH. The EIS is a secure International Health Regulations (IHR) communication database, wherein the WHO Secretariat publishes information about acute public health risks that have potential international implications and provides contact details of other NFPs for direct communication among different states [24]. The NFP also shares reports with the BAI for surveillance of specific diseases and threats. However, outside the DOH and apart from BAI, there is a lack of coordination and communication between the NFP and other agencies. It is, therefore, recommended to develop a national Standard Operating Procedure (SOP) discussing information sharing, field investigation, response, sharing of logistics/resources for food safety incidents, outbreaks, and emergencies [25].

d. Planning and Preparedness

Simulations and training exercises play a crucial role in preparing individuals and organizations for potential health emergencies. These initiatives provide a safe and controlled environment to test response strategies, identify gaps, and enhance coordination among different sectors involved in One Health. In terms of emergency preparedness, the Philippines has established the National Disaster Preparedness Plan 2015–2028, multi-hazard response plans, the Manual of Operations and SOPs for Health Emergency and Disaster Response Management, as well as the Philippine Avian Influenza Protection Program [25]. The government has previously conducted a preparedness simulation exercise on Avian Influenza. Planning and action research programming on field and on-farm disease investigation, surveillance, and control management has also been done through The 1Health Project (“1Health”) for One Human-Environment-Animal Linkage for Total Health [26]. To further support planning and preparedness activities, the Department of Science and Technology (DOST) has launched the first Disaster Risk Reduction Management in Health (DRRM-H) Center in 2022 through the University of the Philippines Manila (UPM) [27,28]. The center seeks to equip emergency responders and local government units with the appropriate skills and knowledge through simulation, as well as by providing tools.

e. Workforce Development

Each sector has plans and initiatives that aim to build the capacity of the workforce in their respective sectors. The Human Resources for Health (HRH) Network of the Philippines is the central body committed to harmonizing policy directions to develop a competent health workforce across the country [29]. With the aim of enhancing the availability of high-quality healthcare services and addressing the unequal distribution of healthcare professionals, the DOH implements the HRH Deployment Program to redistribute the healthcare personnel in the country, aiming to enhance the quality of healthcare services and address the uneven distribution of healthcare professionals. Along with the department's Competency Model and Standards, it also carries out a Learning and Development Plan to identify the gaps in competencies required to fulfill specific job requirements and address them through short courses or formal education. However, the National Human Health Resources for Health Master Plan 2020–2040 which serves as the guideline for the HRH network, is in need of integration of both animal and environmental health plans [30].

Different programs are in place for the development of sector- and field-specific competencies, such as the Philippine Field Epidemiology Training Program (PFETP). The PFETP provides training for primary responders to prepare them for outbreaks by conducting training for surveillance, risk assessment, and response at all levels [31]. There were also efforts in conducting nationwide training on infection prevention and control (IPC) for healthcare workers, regional IPC coordinators, licensing officers, and surveillance offices in 2018 [19]. In the animal sector, veterinary services are supported by training from the BAI, Agricultural Training Institute (ATI) and local government units (LGUs). The ATI was specifically established to increase the capacity of workers in the agriculture and fisheries sector by offering scholarships, extension programs, and training [32]. In the environmental sector, the Philippine Green Jobs Act (Republic Act No. 10771) [33] was enacted to support the country's transition towards a sustainable economy, promote the creation of green jobs, and develop the skills and capabilities of the workforce related to green jobs through training programs. As defined by the International Labour Organization (ILO), green jobs refer to decent (i.e. safe working conditions and adequate wages) employment opportunities that contribute to environmental conservation, such as agricultural scientists, ecologists, and conservation officers [34,35]. The DENR also offers competency development programs such as Environment and Natural Resources Academy (ENRA) courses to address knowledge gaps, enhance comprehension of environmental laws, and improve the skills of DENR employees [36]. However, during the Environmental Health Needs Assessment and Validation Workshop held last April 2023, DENR assessed that there is no established in-service training program for environment professionals to contribute to One Health.

f. Risk Communication

Risk communication aims to promote awareness and knowledge of risks while encouraging health-protective practices among populations and sectors of society [37]. The Communications Office (COM) under the Office of the Secretary is responsible for handling the risk communication plans in the DOH. To relay information to the public, the DOH has officers in the Centers for Health Development (Regional Office) as the regional counterparts of the COM office, as well as assigned spokespersons for both the national and regional tiers. The COM coordinates with other relevant bureaus as part of their responsibility to generate and relay the necessary information when outbreaks and emergencies occur - whether this be through traditional media, social media, quad media, etc. They coordinate with the Media Relations Unit to generate media and social media products, while incorporating analytics to improve their coverage (AO 2017–0007) [38]. In the DA and DENR however, there is no specific bureau assigned for risk communication. Instead, a risk communication plan is developed per program. As an example, the DA has created a strategy when handling cases of African Swine Fever (ASF) - as a part of the National ASF Prevention and Control Program. This strategy primarily focuses on the prevention of ASF by targeting stakeholders who come into contact with swine - namely consumers, travelers, hunters, vendors, hog traders, and veterinarians. Online media, as well as traditional media, is the primary medium of communication for these stakeholders to enable a wide reach for preventing the spread of ASF [39]. The DA has also collaborated with the DENR with regards to risk communication for AI. As part of risk communication, DENR representatives regularly participate in media appearances, such as radio interviews, to communicate with the general public. Finally, other efforts with regards to risk communication is the promotion of public health education to include One Health issues, such as zoonosis or AMR.

## 4 Discussion

This study highlights several critical issues and recommendations related to the technical activities of the Multisectoral One Health coordination framework. Strengthening governance mechanisms that ensure the implementation of PhilCZ activities and empower the involvement of LGUs are necessary to improve the implementation of One Health efforts in the Philippines. It is essential to enhance accountability and stewardship of LGUs through optimized resource allocation, IHR awareness at all levels, and innovative institutional mechanisms with improved oversight. Multi-sectoral policy instruments for the joint implementation programs as well as allocation of budget for these activities are also crucial factors that may improve the operationalization of One Health activities. Reliable and sustained financing is crucial to continue One Health activities and expand these to other issues beyond zoonotic diseases [40]. The development of a “One Health Act” legislation is suggested, along with the creation of One Health Response Teams and resource allocation and mobilization mechanisms at national, regional, and local levels. Priorities, commitment and budget allocation for One Health implementation efforts should be aligned, as well as the creation of a budget tracking and monitoring framework for regular sources of funding.

Successful multi-sectoral collaboration is dependent on a shared vision, trust, and credibility, which are built in both positive and difficult situations with fairness and transparency [41,42]. Without clearly defined and delineated leadership roles, information, and structure, there will be fragmented implementation and loose accountability. This also necessitates a carefully cultivated environment across levels, as achievements at one level do not necessarily guarantee success at lower tiers [43]. These bottlenecks must be identified to increase efficiency and effective implementations [42]. At present, the execution of One Health initiatives relies on the capacities of personnel drawn from various departments, resulting in a disjointed approach and excessive workload. Departments often have different, although overlapping, priorities due to lack of efficiency and utilization of existing governance mechanisms [42]. Trained and dedicated One Health Response Teams would facilitate unified implementation of these endeavors, however, the creation of a separate agency must be approached with careful consideration as it may become redundant or dilute the current functions of the tripartite agencies. Our results also revealed a notable bias towards addressing zoonotic diseases across the technical activities, sidelining crucial environmental concerns and climate change issues, such as tackling antimicrobial resistance (AMR) in wastewater, food safety, non-communicable diseases, climate changes [40,41]. The focus of One Health initiatives must be expanded to encompass a wider range of issues under a shared vision and priority areas.Toolkits in mapping stakeholders, developed by the international organizations and academic institutions, have proven useful in the analysis and revision of One Health governance mechanisms and existing protocols in other countries and should be considered for adoption in the Philippine context [40,44,45].

The results of the review emphasize the use of good practices and lessons learned from health responses to create guidelines and improve action on medical countermeasures and personnel deployment. Active One Health surveillance and the development of a Joint One Health National Disease Surveillance, Prevention, and Control System within 24-h response time are recommended. Joint risk assessment, surveillance, and information sharing need to be streamlined across sectors, and data quality, timeliness, and completeness must be enhanced.

Joint risk assessment, surveillance, and information sharing in the country is limited by the gaps listed in the Annex. In spite of the support given by the DOH to monitor human health and the DA to monitor animal health, risk assessments lack mechanisms for joint surveillance and information sharing. Risk assessment and surveillance mechanisms operate in silos, with sharing protocols in place; however, integration into mainstream practices is lacking, leading to delayed responses and non-real-time data dissemination. Moreover, the implications of data or interpretations across sectors remain unclear, impeding the optimization of resources for collaborative investigation and response efforts. It is crucial to enhance collaboration and coordination among relevant stakeholders, including government agencies, healthcare institutions, research organizations, and other community representatives. One review revealed the importance of both formal and informal communication in joint One Health investigations and response [40].

Existing networks and links should be strengthened between points of entry (POE), healthcare facilities, and public health systems in both human and animal settings to facilitate a streamlined surveillance protocol and rapid reporting of events. Investing in the development of testing capabilities and sampling coverage is essential. This involves increasing sampling coverage, optimizing systems, guidelines, and methodologies for health emergency assessments. Lastly, the operationalization of a joint risk assessment involving the different stakeholders at all levels, for both human and animal samples, is crucial in proactively identifying and managing risks, thus improving overall public health outcomes. A Joint One Health National Disease Surveillance, Prevention, and Control System with a timely response system can be established, as well as technical working groups with representatives from all levels. As most infectious disease outbreaks occur first at the community-level, leveraging technology and electronic data sharing to connect local information to national and global networks in a timely manner has been proven effective in the mitigation and control of such public health concerns [40,46]. This concerted effort ensures the formulation of proactive strategies and policies aimed to protect public health, animal welfare, and the overall ecosystem, ultimately fostering a harmonious coexistence between those situated within the country.

Several programs primarily highlight the country's efforts in fostering collaborative response efforts. To facilitate these programs, interagency collaborations through the ICAMR, PhilCZ, NDRRMC, Food Safety Regulation Coordinating Board (FSRCB), and Inter-Agency Task Force for the Management of Emerging Infectious Diseases in the Philippines (IATF-EID) have been established to address AMR, zoonoses, disasters, food safety, and EIDs respectively. At the national planning and policy level, the Philippines has actively participated in various global One Health initiatives spearheaded by tripartite organizations. These include workshops such as the JEE workshop, IHR workshop, National Bridging Workshop (NBW), and One Health zoonotic disease prioritization (OHZDP) [47]. The country has developed its own national action plans against antimicrobial resistance (AMR) [48], aligning with the Global Action Plan on Antimicrobial Resistance established in 2015 and have committed to the implementation of programs like the Tricycle surveillance program in both the human and animal sectors [49]. Food safety regulations in the Philippines are guided by the Codex Alimentarius Commission, with a National Codex Organization to respond to Codex Related issues and concerns [50]. While the Philippines' engagement in these initiatives is robust, there remains room for enhancement, particularly in refining implementation strategies and streamlining coordination and information sharing at the regional and local levels.

The aforementioned issues are addressed through the implementation of issue-specific investigations and assessments and the development of guidelines and frameworks. A national action plan is in place to contribute to AMR control and prevention measures [48]. IPC has also been further strengthened through the National Standards and Policy, the establishment of a National Technical Working Group, and mandatory IPC licensing for hospitals [25]. Zoonotic diseases are addressed through the activities of PhilCZ and the emerging and re-emerging infectious diseases (EREID) program. The food regulatory system in the country is reinforced through the Food Safety Act of 2013. Effective monitoring is observed, with food-borne diseases regularly reported and laboratory testing for human specimens and food samples.

Limitations in resources, capacity, and infrastructure have been identified as gaps in joint investigation and response that leads to limited scope and coverage of related activities. As such, further system strengthening and capacity-building are required to ensure that multi sectoral activities of the stakeholders are conducted effectively [19]. International organizations are also important stakeholders in One Health, as they aid in providing financial support and human resources for One Health-related activities [40]. For example, the tripartite organizations have disseminated the One Health operational framework across WHO member states and have remained committed to providing technical support to help other countries reach their International Health Regulation (IHR) obligations [51]. Building capacity for outbreak response is a critical component of this technical area. This involves strengthening systems, services, and monitoring capabilities to detect, respond to, and mitigate health threats effectively.

In terms of workforce development, there have been on-going initiatives for training, research, and educational opportunities to capacitate the One Health workforce. The Field Epidemiology Training Program (FETP) of the DOH [52] and the Applied Veterinary Epidemiology Training (AVET) and ASF Preparedness Workshop of the DA [53] both train field epidemiologists to conduct investigations during outbreaks, however a noted challenge is that epidemiologist positions are not formalized on the salary grade list. The country is also a member of the Southeast Asian One Health University Network (SEAOHUN), established with the support of USAID, that aims to provide a resilient and trained One Health workforce with its member universities actively participating in fellowship opportunities [54]. However, existing training opportunities are often short-term and informal (i.e. online classes, workshops). There is potential for improvement by establishing a more formalized curriculum within the education and research system. Moreover, offering tangible job prospects can serve as a catalyst for attracting greater student interest. There is a need to capacitate the technical skills of personnel through the conduct of joint training programs, build data analysis and quality management skills, and improve the awareness and understanding for the concept of One Health. Existing One Health training programs should be geared towards detecting, reporting, assessing, managing, and responding to diseases.

Through the aforementioned efforts, it is evident that there is a need for a more robust risk communication training and plan for community engagement across the different sectors. Community engagement is particularly important to contextualize issues on a local level which then facilitate behavior change, especially AMR behavior change [55]. Community-based initiatives play a crucial role in ensuring that programs are not only community-owned but also rooted in robust evidence, equity, and inclusivity. By prioritizing local needs and preferences over donor priorities, these efforts empower communities, foster a sense of ownership, and ultimately lead to more sustainable and impactful outcomes [46]. It is also encouraged that a risk communication program be established, which will include the implementation and evaluation of these efforts. Moreover, it is important that the risk communication program that is to be developed employs a framework for routine evaluation of planned risk communication interventions and testing of operational mechanisms. Finally, there is a lack of a coordination mechanism and an established formalized commitment of partner sectors. It is recommended that key sectors should be strengthened; spotlighting trade and industry, plant, aquaculture, education and local governments.

### 4.1 Limitations of the study

This study has some limitations, including limited access to recent but unpublished institutional papers and a scarcity of peer-reviewed publications on One Health at the local level. Nevertheless, the study's findings remain valid and will play a crucial role in shaping future research, policies, and practices related to the implementation of One Health approaches in the Philippines. These limitations highlight the ongoing need for further research and documentation of local One Health initiatives to bolster the evidence base for future endeavors.

## 5 Conclusion

In the aftermath of the COVID-19 pandemic, as the world transitions to a new normal, the imperative for a strategic plan to fortify human, animal, and environmental health systems against the threat of emerging and re-emerging diseases has never been more critical. This study underscores the urgent need for enhanced governance, multi-sectoral collaboration, and strategic investments within the Philippines' Multisectoral One Health framework, advocating for a unified approach to comprehensively address public health, animal welfare, and ecosystem integrity. It calls for the creation of a “One Health Act,” establishment of One Health Response Teams, and targeted funding mechanisms, alongside improved local government unit involvement and accountability. Additionally, the Pandemic Accord, which is an ongoing development by the WHO, identifies One Health as a key approach in the management of future public health emergencies. If ratified as a treaty, governance mechanisms and resource mobilization may be streamlined to more equitable access across sectors when addressing future pandemics and other global emergencies [[56], [57]]. Highlighting the necessity to streamline One Health initiatives and improve coordination to eliminate redundant efforts, the study stresses the importance of closing the gap in integrated surveillance and information sharing across health sectors. It also identifies the need for capacity building, technical training, and the development of a robust risk communication strategy to enhance disease detection, reporting, and manage antimicrobial resistance effectively. Despite the increase in government and non-government support for One Health initiatives, significant deficiencies in joint investigation, response capabilities, and coordination mechanisms remain, emphasizing the critical role of sectoral partnerships in ensuring a comprehensive multisectoral action for health and environmental protection.

## Ethics approval

The Ateneo de Manila University Research Ethics Committee waived the need for ethical approval and the need to obtain consent for collection, analysis, and publication of secondarily, retrospectively obtained for this non-interventional study. All methods were performed in accordance with the relevant guidelines and regulations.

## Consent for publication

Not applicable.

## Funding

The study was funded by the 10.13039/100004423World Health Organization.

## CRediT authorship contribution statement

**Lystra Zyrill A. Dayapera:** Data curation, Formal analysis, Investigation, Resources, Supervision, Validation, Visualization, Writing – original draft, Writing – review & editing. **Jenica Clarisse Y. Sy:** Data curation, Formal analysis, Investigation, Methodology, Validation, Visualization, Writing – original draft, Writing – review & editing. **Sary Valenzuela:** Conceptualization, Data curation, Formal analysis, Funding acquisition, Investigation, Methodology, Project administration, Resources, Supervision, Writing – original draft, Writing – review & editing. **Samantha Julia L. Eala:** Data curation, Formal analysis, Investigation, Writing – original draft, Writing – review & editing. **Ciara Maria Ines P. Del Rosario:** Formal analysis, Writing – original draft, Visualization. **Karen Nicole C. Buensuceso:** Data curation, Formal analysis, Writing – original draft. **Adrian S. Dy:** Formal analysis, Visualization, Writing – original draft. **Danielle A. Morales:** Funding acquisition, Investigation. **Anna Giselle Gibson:** Supervision. **Geminn Louis C. Apostol:** Funding acquisition, Supervision, Writing – original draft.

## Declaration of competing interest

The authors declare that they have no competing interests.

## Data Availability

The policies and documents included in the study were acquired with permission from the Philippines Department of Health Epidemiological Bureau, Department of Environment and Natural Resources Biodiversity Management Bureau, Department of Agriculture Bureau of Animal Industry. The policies and documents may be accessed publicly on their websites or upon formal request from the corresponding author or by contacting the data source owners directly at eb@doh.gov.ph, bmb@bmb.gov.ph, and director@bai.gov.ph respectively.
